# Speeding Up the Discovery of Optimal Feature Combinations for Omics Data Based on Pseudo-Kernel Function

**DOI:** 10.21203/rs.3.rs-9864895/v1

**Published:** 2026-06-18

**Authors:** Shipeng Ren, Guoqing Yang, Deyin Yu, Anqi Wang, Qiwei Li, Sijia Zhang, Chao Li

**Affiliations:** * College of Information Engineering,; a Dalian Key Laboratory of Smart Fisheries, Dalian 116023, Liaoning Province, P. R. China; b Dalian Ocean University, Dalian 116023, Liaoning Province, P. R. China

**Keywords:** Omics data analysis, Feature combination, Kernel function, Computational cost, Pathways in cancer

## Abstract

Discovering meaningful feature (molecule) combinations to define simple, accurate, and easily interpretable decision rules for disease classification and prediction can improve the study of disease diagnosis and prognosis. However, the computational time complexity of constructing feature combinations for each feature pair in existing methods is often problematic or prohibitive, as the number of features is often in the order of tens of thousands. To significantly reduce the computational cost and maintain the classification performance, this paper proposed a novel acceleration algorithm and a new omics data analysis method based on pseudo kernel functions (PKF-k-TSP). PKF-k-TSP explores the linear and nonlinear combination of features by pseudo kernel function, evaluate feature interaction, and selects k>0 top-scoring pairs to build an ensemble classifier. PKF-k-TSP maps feature pairs from a low-dimensional space to a high-dimensional feature space by a mapping function, as the same effect as kernel function, and ensure the classification performance. However, it significantly reduces the computational time costs. Experimental results demonstrate that PKF-k-TSP achieves superior classification performance, while exhibiting significantly improved computational efficiency compared with KF-k-TSP, with the running time reduced by 72.43%. Furthermore, the feature pairs identified by PKF-k-TSP align with physiological and pathological changes, offering insights into disease mechanisms. The method also excels in cross-cancer pathway interaction analysis, capturing both conserved and tissue-specific signaling networks. Hence, PKF-k-TSP enables rapid and efficient feature mining, which is especially suitable for large-scale disease omics data analysis.

## 1. Introduction

With the development of life omics experimental techniques, a deluge of various omics data has emerged (Li et al. 2023b). The challenge lies in how to discover potential biomarkers from disease omics data to achieve diagnosis and treatment of diseases such as cancer, which has become a research hotspot in systems biology (Rattray et al. 2018). However, the characteristics of high dimensionality and small sample size in disease omics data pose challenges for the discovery of potential biomarkers (Withnell et al. 2021). Recently, many biomarker discovery methods have been developed and applied in disease omics data to mine the information reflecting the nature of physiological and pathological changes in living organisms, such as Support Vector Machine (SVM) (Gregoire et al. 2025), Analysis of Variance (ANOVA) (Eun et al. 2018), least absolute shrinkage and selection operator (LASSO) (Usai et al. 2009), Random Forest (RF) (Tang et al. 2018)and Gradient Boosting Machines (GBM) (Jose et al. 2025), etc. However, these methods focus on identifying molecular biomarkers to distinguish different disease stages, selecting an excessive number of features (biomolecules), which leads to the classification model complex and less interpretable (Mohamed et al. 2024). Concurrently, the advancement of deep learning has introduced novel methodologies for omics data analysis, enabling dimensionality reduction of gene expression data for identifying cancer-specific biomarkers (Lin et al. 2024; Han and Anne 2025). Although the advanced deep learning algorithms can deal with high-dimensional omics data, the deep learning model is a “black box”, which is difficult to ensure the interpretability of models (Behrmann et al. 2018; Zhao et al. 2025). Hence, developing a simple, efficient, and interpretable biomarker discovery and disease classification method is preferred for analyzing the high-dimensional, small-sample omics data (Rudin 2019).

To address the limitations of complexity and interpretability, the methods focusing on feature combinations have gained attention. Geman et al. (2004) proposed the top scoring pair (TSP) method, which selects feature pair(s) based on expression differences, offering simple, competitive, and biomedically interpretable classification rules Recognizing TSP's limitation of selecting only one pair, later advancements, such as k-TSP and TST, extended this by selecting multiple top-ranking disjoint pairs.Huang et al. (2018) focused on feature vertical and horizontal relationships simultaneously and proposed VH-k-TSP, which defined the important feature pairs and builds a hybrid classifier. Meanwhile, TSP and k-TSP (Ene et al. 2016) examine each pair in the same fixed pattern(the “x-y=0” line in 2D space), they ignored the general linear forms of feature combinations. Our previous works further explored feature combinations: LC-k-TSP (Lin et al. 2019) incorporated linear relationships, and KF-k-TSP (Li et al. 2023a) extended this to simultaneously explore linear and nonlinear combinations and allows hybridization of multiple kernel functions for comprehensive feature interaction evaluation. While these TSP-family algorithms offer high interpretability and strong classification performance by focusing on feature relationships, their major drawback lies in the prohibitively high computational complexity when constructing and evaluating combinations for every feature pair in high-dimensional omics data.

Specifically, the computational times for all feature pairs is n(n-1)/2, where n is the number of features (biomolecules) (Luo et al. 2025). The computational time complexity of TSP method is $O\left(mn^{2}\right)$, including m is the number of samples. The time cost of LC-k-TSP method is $O\left(2*m^{2}n^{2}\right)$, the time cost of KF-k-TSP method with two kernel function is $O\left(4*m^{2}n^{2}\right)$. However, one characteristic of omics data is “high dimensions, small samples”, which have a large number of features but few samples. The computation time of TSP family algorithms increases exponentially with the increase of the feature dimension (Zhou et al. 2020). Meanwhile, to ensure the classification performance and generalization ability of an algorithm, a cross-validation process is typically employed to train the model, which further increases computational cost. This challenge is further exacerbated by necessary cross-validation procedures for robust model evaluation (Leek et al. 2010; Squillante 2014). While some acceleration algorithms exist (e.g., RNGCS (Khamesipour and Kagaris 2019) or filtering methods like our previous work based on Interaction Gain (Jin et al. 2023)), they often reduce computation by pre-filtering features, which risks significant information loss and suboptimal model performance (Fan et al. 2011). Therefore, there is a critical need for effective acceleration algorithms that can expedite the evaluation and selection of feature pairs across the entire feature space while preserving optimal model performance (Wang 2022).

In this study, we revisit feature-pair interaction modeling from a biological and methodological perspective. Rather than treating kernel functions solely as mathematical tools for nonlinear transformation, we argue that explicit low-order interaction representation provides a more appropriate and biologically grounded modeling paradigm for high-dimensional omics data. In many molecular regulatory systems, disease-associated effects are primarily driven by pairwise or low-order cooperative interactions, whereas high-order kernel expansions often introduce redundant representations, increased computational burden, and limited interpretability. From this viewpoint, we propose PKF-k-TSP, an explicit interaction-based feature combination framework that models both linear and nonlinear relationships through a compact pseudo-kernel mapping. By projecting each feature pair into a low-dimensional interaction space spanned by $\left\{x_{1},x_{2},x_{1}x_{2}\right\}$, PKF-k-TSP preserves the essential expressive power required for capturing biologically meaningful interactions while avoiding the instability and inefficiency associated with implicit high-order kernel functions. This design enables a principled balance between interaction modeling capability, computational efficiency, and interpretability, which is particularly critical for small-sample, high-dimensional omics data analysis.

Table 1 summarizes the statement of significance required by the journal.

## 2. Methods

### 2.1. Kernel function

Support vector machine(SVM) is one of the-known machine learning algorithms, which is widely applied in omics data analysis. The base idea of SVM is to separate different class labels by finding a hyper-plane $w^{\text{T}}x+b=0$ with maximum-margin by objective function (See Equation (1)) (Chang and Lin 2011). When the omics data is linearly separable, SVM achieves competitive results.


(1) $$\underset{\omega ,b}{\text{min}}\frac{\|\omega {\|}^{2}}{2}\;\text{s.t.}\;y_{i}\left(\omega^{T}x_{i}+b\right)\geq 1,\;i=1,2,\cdots ,m$$

Equation (1) represents the quadratic programming problem, which can be formalized into Lagrange formula by combing the objective function and the constraint as follows:

(2) $$\text{min}\;L_{P}=\frac{\|\omega {\|}^{2}}{2}-\sum_{i}a_{i}\left(y_{i}\left(\omega^{T}x_{i}+b\right)-1\right)\;=\frac{\|\omega {\|}^{2}}{2}-\sum_{i}a_{i}y_{i}\left(\omega^{T}x_{i}+b\right)+\sum_{i}a_{i}$$

Where $a_{i}$ is the Lagrange multiplier. To solve an objective function, the values of w, b and a are calculated by computing the partial derivatives of objective function and setting the derivative equal to zero as follows:

(3) $$\frac{\partial L_{P}}{\partial w}=0\Rightarrow w=\sum_{i=1}^{m}a_{i}y_{i}x_{i}\;\frac{\partial L_{P}}{\partial b}=0\Rightarrow \sum_{i=1}^{m}a_{i}y_{i}=0$$

Hence, the objective function $L_{P}$ can be written as the dual form $L_{D}$ as follows:

(4) $$\text{max}\;L_{D}=\sum_{i}a_{i}-\frac{1}{2}\sum_{i,j}a_{i}a_{j}y_{i}y_{j}x_{i}^{T}x_{j}\;\text{s.t.}\;\sum_{i=1}^{m}a_{i}y_{i}=0,a_{i}\geq 0,i=1,2,\cdots ,m$$

The values of w, b and a can be determined by solving the Equation (3) (Cortes and Vapnik 1995) and Equation (4) (Apsemidis et al. 2020). However, for the nonlinearly separable omics data, a nonlinear mapping function(ϕ) can be used to transform data into a higher-dimensional feature space, where the omics data can be linearly separable. The objective function (Equation 4) of SVM will be rewritten as follows:

(5) $$\text{max}\;L_{D}=\sum_{i}a_{i}-\frac{1}{2}\sum_{i,j}a_{i}a_{j}y_{i}y_{j}\phi {\left(x_{i}\right)}^{T}\phi \left(x_{j}\right)\;\text{s.t.}\;\sum_{i=1}^{m}a_{i}y_{i}=0,a_{i}\geq 0,i=1,2,\cdots ,m$$

**Fig. 1:**
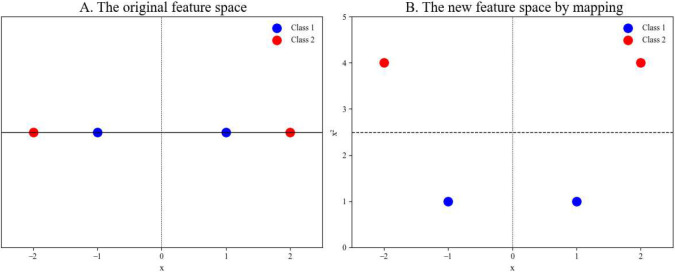
A simple example of feature transformation. A. The original feature space. B. The new feature space by mapping.

For example, a nonlinear separable data contain two features $\left(x_{1},x_{2}\right)$ and two classes. The mapping function maps the data from the input space $\left(X\in R^{2}\right)$ to a high-dimensional space $\left(F\in R^{3}\right)$ where a linear separation is obtained. To illustrate this concept more intuitively, a simple example is shown in Figure 1. Suppose two classes of data points are given: Class 1 with samples at (x=1) and (x=-1), and Class 2 with samples at (x=2) and (x=-2). In the original input space, as shown in Figure 1A, the original feature space is non-linearly separable. However, by applying a nonlinear mapping function $\phi (x)=x^{2}$, the original feature space $\left(\{x\}\in R^{1}\right)$ is transformed into a higher-dimensional feature space $\left(\left\{x,x^{2}\right\}\in R^{2}\right)$ as follows: ϕ(1)=ϕ(-1)=1, and ϕ(2)=ϕ(-2)=4. This transformation projects the data into a new space where Class 1 is mapped to (1, 1), (−1, 1) and Class 2 to (2, 4), (−2, 4), enabling linear separation, as illustrated in Figure 1B. This example clearly demonstrates how nonlinear transformations can project inseparable data into a feature space where linear separation becomes possible.(see Fig.1)

Due to high-dimensional features, it is difficult to compute $\phi {\left(x_{i}\right)}^{T}\phi \left(x_{j}\right)$ directly. Hence, kernel function $K\left(x_{i},x_{j}\right)$ can be introduced to avoid complex computation. The kernel function is defined as the dot product of nonlinear mapping functions as follows:

(6) $$K\left(x_{i},x_{j}\right)=\phi {\left(x_{i}\right)}^{T}\phi \left(x_{j}\right)$$

The common kernel functions are linear kernel function $\left(K\left(x_{i},x_{j}\right)=x_{i}^{\text{T}}x_{j}\right)$, polynomial kernel function $\left(K\left(x_{i},x_{j}\right)={\left(\gamma {x_{i}}^{\text{T}}x_{j}+c\right)}^{d}\right)$ and RBF kernel function $\left(K\left(x_{i},x_{j}\right)\right.\left.=\text{exp}\left(\gamma {\left\|x_{i}-x_{j}\right\|}^{2}\right)\right)$ (Wang et al. 2015; Galal et al. 2022; Danieli et al. 2023).

### 2.2. PKF-k-TSP algorithm

To improve and speed up the modeling capability of the KF-k-TSP algorithm for the potential linear and nonlinear relationships between features, this paper proposes an improved algorithm, PKF-k-TSP. PKF-k-TSP introduces multiple explicit combination mapping functions ϕ(x) for each pair of features $\left(x_{1},x_{2}\right)$, maps the original two-dimensional feature space to a new higher-dimensional feature space, which can enhance the classifier's ability to capture complex patterns. Our previous method KF-k-TSP used two kernel functions (the Linear kernel function and the polynomial kernel function) to explore feature combinations from linear and nonlinear views perspectives and achieved better performance than other TSP family methods. The polynomial kernel function is used to map data from the original feature space to a higher-dimensional space and effectively capture the nonlinear relationships between data points. The typical forms of a polynomial kernel are as follows (Zhang 2001):

(7) $$K_{1}\left(x_{1},x_{2}\right)={\left(x_{1}^{T}x_{2}\right)}^{d}$$

(8) $$K_{2}\left(x_{1},x_{2}\right)={\left(x_{1}^{T}x_{2}+c\right)}^{d}$$

These forms correspond to explicit mappings that expand the input features into polynomial combinations of all degrees up to d, where d denotes the polynomial degree, and c is a constant that influences the contribution of higher-order terms. Building on this concept, this paper introduces five mapping functions based on binary feature combinations, each corresponding to a distinct form of polynomial kernel expansion. Each mapping function is used as a substitute for the kernel function, named pseudo-kernel function, to construct the model. We will introduce in detail the relation between the mapping functions and kernel functions as follows. The relationship between the mapping functions and kernel functions is introduced below.

When the degree(d) of the polynomial kernel function is one, d=1, in Formula 7 and Formula 8 this form corresponds to an approximate expansion of the feature combination of the linear kernel function $K\left(x,x^{\prime }\right)=x^{T}x^{\prime }$. The feature space is spanned by $\left\{x_{1},x_{2},1\right\}$. It is easy to see that the polynomial kernel of degree 3 in Formula 7 corresponds to a feature space spanned by all products of 2 variables, that is $\left\{{x_{1}}^{3},{x_{2}}^{3}\right.,\left.x_{1}^{2}x_{2},x_{1}x_{2}^{2}\right\}$. The polynomial kernel of degree 2, constant 1 in Formula 8 corresponds to a feature space spanned by all products of 2 variables, that is $\left\{1,x_{1},x_{2},{x_{1}}^{2},{x_{2}}^{2},x_{1}x_{2}\right\}$. It is easy to see that the polynomial kernel of degree 3 in Formula 8 corresponds to a feature space spanned by all products of 2 variables, that is $\left\{1,x_{1},x_{2},{x_{1}}^{2},{x_{2}}^{2},x_{1}x_{2},{x_{1}}^{2}x_{2}\right.,x_{1}{x_{2}}^{2},{x_{1}}^{3},{x_{2}}^{3}\}$. Based on this idea, this paper investigates kernel function transformations by mapping function, aims to improve feature interaction modeling and optimize computational efficiency.

The five kernel functions introduced above include the basic first-order interaction pattern (corresponding to the case of d=1), extend to the quadratic and cubic polynomial kernel functions $K_{1}$ and $K_{2}$, which can capture feature interactions at different levels by variable products in their feature spaces. The first-order interaction pattern has remarkable advantages. It, along with other transformation forms, belongs to the system of constructing feature spaces via variable products, yet it is the simplest. For instance, ${(x}_{1}x_{2})$ can directly demonstrate variable interactions, efficiently capture key feature interactions. Higher-order polynomial kernels can build complex feature spaces, but the cumbersome calculations lead to a sharp increase in time complexity and are prone to overfitting. Essentially, the complex feature interactions of higher-order polynomial kernels are developed based on simple forms like the first-order interaction pattern. The first-order interaction pattern not only embodies the fundamental principles of feature interactions but also stands out for its high efficiency and reliability in practical applications, being the most advantageous option. Hence, our method PKF-k-TSP introduces the mapping function and transforms feature pairs from two-dimensional space to three-dimensional space spanned by $\left\{x_{1},x_{2},x_{1}x_{2}\right\}$. Then, PKF-k-TSP explores the linear combinations and determine the best combination form in the new three-dimensional space. At last, PKF-k-TSP selects the k top-scoring feature combinations to build the classifier.

Let *PseudoKernel* be the pseudo kernel function, *XTrain* be the training dataset, *XValidation* be the validation dataset, and k be the number of selected feature pairs. For each feature pair, PKF-k-TSP uses the mapping function in *PseudoKernelSet* to generate a new feature space and combination features. Then, PKF-k-TSP constructs SVM model on training data *XTrain* using a linear kernel in the new feature space and scores evaluates the new combination features by the largest classification performance of trained an SVM on *XValidation*. Next, PKF-k-TSP ranks all feature pairs based on their scores in descending order. Finally, PKF-k-TSP selects the best k top-scoring feature pairs and obtains an ensemble classifier based on the k top-scoring pairs.

The details of the PKF-k-TSP algorithm is presented in Algorithm 1.

**Algorithm 1.** PKF-k-TSP algorithm

**Input:** *XTrain, XValidation*, $F=\left\{f_{1},f_{2},\ldots ,f_{p}\right\}$, *PseudoKernel, and* k.
**Ouput:** *The selected feature pairs set SelFP and the ensemble classifier MPKF*.
**Initialization:** SelFP=ϕ,
**Begin**
For each pair of features $\left(f_{i},f_{j}\right)$ where 1<=i<j<=p DO:
*XTrain_mapped* $\left(f_{i},f_{j}\right)$ = Transform the feature pair $\left(f_{i},f_{j}\right)$ form *XTrain* into new feature space by pseudo kernel function in *PseudoKerne*;
*XValidation_mapped* $\left(f_{i},f_{j}\right)$ = *Transform the feature pair $\left(f_{i},f_{j}\right)$ form XValidation into new feature space by pseudo kernel function in PseudoKerne*;
Construct an SVM model $SVM_{ij}$ with linear kernel function based on *XTrain_mapped* $\left(f_{i},f_{j}\right)$;
Obtain the classifiction accuracy rate *acc* of $SVM_{ij}$ on *XValidation_mapped* $\left(f_{i},f_{j}\right)$;
Score(i,j)=acc;
Rank all the feature pairs according to their *scores Score* in descending order;
$\text{SelFP}=\left\{\text{The}\;k\;\text{top-scoring}\;\text{pairs}\right\}$
*MPKF* = Ensemble classifier consisting of the k best SVM classifiers corresponding to the k top-scoring pairs;
Return the classifier *MPKF*, *SelFP*;
**End**

## 3. Results

To validate the effectiveness of the PKF-k-TSP method, we firstly conducted a comparative analysis between PKF-k-TSP and KF-k-TSP from classification accuracy, time complexity, computational cost, and feature selection consistency. Then, we systematically evaluated the performance of the PKF-k-TSP algorithm on 8 public datasets. By presenting the experimental results for odd values of k in each dataset, we highlighted the advantages of this algorithm in feature space transformation. Meanwhile, PKF-k-TSP was compared with five transformation strategies, and four TSP family algorithms to verify its effectiveness. Finally, we validated the biological significance of PKF-k-TSP through an in-depth analysis of lung cancer biomarkers and a broader pan-cancer functional study.

### 3.1 Omics dataset

The experiments utilized eight public datasets to validate the performance of our method PKF-k-TSP, which not only contained different omics data (transcriptomic and metabolomics) but also included different disease types (cancer, parkinsons, and depression). These datasets exhibit substantial variability in both the number of molecular features (ranging from 22 to 556) and sample sizes (ranging from 44 to 569). Specifically, the *Breast cancer* dataset, containing metabolomics profiles, was retrieved from the supplementary materials (Budczies et al. 2013). The *GSE*28700 dataset, a transcriptomic data for gastric cancer, is available from the GEO database (Tseng et al. 2011). Three datasets(*Parkinsons, Promoter*, and *Wdbc*) were obtained from the UCI Machine Learning Repository, which is a standard source for benchmarking machine learning algorithms (Elghazel and Aussem 2015; Markelle et al. 2023). In addition, metabolomics data for *Depression*1, *Depression*2, and *Lung cancer* were sourced from the NIH Common Fund's Metabolomics Data Repository and Coordinating Center (supported by NIH Grant U01-DK097430). These datasets can be accessed via the Metabolomics Workbench portal (http://www.metabolomicsworkbench.org), and are identified by the project IDs ST000062, ST000063, and ST000385, respectively. Table 2 gives the detailed information of the eight datasets. Table 1 summarizes the detailed information of the eight datasets.

### 3.2 Experimental settings

**Fig. 2:**
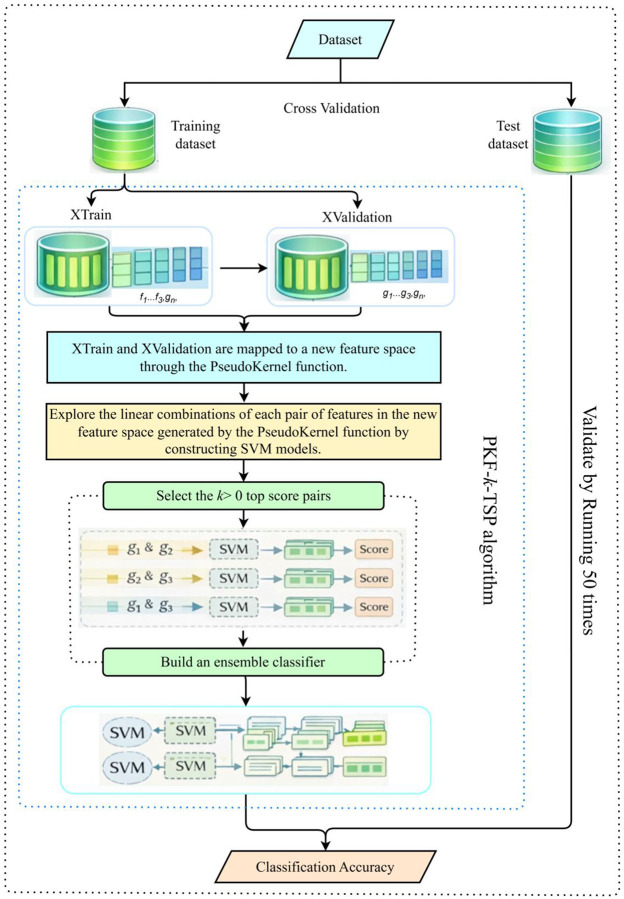
The experimental scheme.

In this experiment, the SVM model with linear kernel function is used as classifier to evaluate performance. Five-fold cross-validation was run 50 times to get the average classification accuracy rate of each method. The value of parameter k can be set based on the experience or be determined by an inner cross-validation. The primary objective of the experiment is to assess the performance of the PKF-k-TSP algorithm for different fixed values of k, specifically k=1,3,5,7,9,11,13, and 15 (for the Parkinsons dataset, the maximum k setting was 11 due to the fewer number of features) 7. Subsequently, the PKF- k-TSP method, with the k setting determined by an inner cross-validation, was compared against the benchmark methods. Figure 2 illustrates the experimental design for the PKF-k-TSP algorithm. Regarding computational costs in cross-validation, the linear kernel SVM is computationally efficient as it avoids constructing complex kernel matrices.

PKF-k-TSP utilizes the mapping function to transform the two-dimensional feature space into a new high-dimensional space. The form of the input mapping functions can directly affect the computation cost and the performance. Hence, we discussed the different mapping functions in the experiment, and tested other four kinds of kernel function settings, named PKF-k-TSP_1_, PKF-k-TSP_2_, PKF-k-TSP_3_ and PKF-k-TSP_4_, which represent the types of mapping function (|*PseudoKernelSet*|) in PKF-k-TSP. The detailed descriptions of kernel functions and mapping functions **are** shown in Table 3. Our method PKF-k-TSP uses the mapping function to generate the new feature space spanned by $\left\{x_{1},x_{2},x_{1}x_{2}\right\}$. PKF-k-TSP_1_ represents PKF-k-TSP method with the spanned feature space $\left\{{x_{1}}^{2},{x_{2}}^{2},x_{1}x_{2}\right\}$ in *PseudoKernelSet* based on the kernel function $K\left(x_{1},x_{2}\right)={\left({x_{1}}^{\text{T}}x_{2}\right)}^{2}$. PKF-k-TSP_2_ represents PKF-k-TSP method with the spanned feature space $\left\{1x_{1},x_{2},{x_{1}}^{2},{x_{2}}^{2},x_{1}x_{2}\right\}$ in *PseudoKernelSet* based on the kernel function $K\left(x_{1},x_{2}\right)={\left(1+{x_{1}}^{\text{T}}x_{2}\right)}^{2}$. PKF-k-TSP_3_ represents PKF-k-TSP method with the spanned feature space $\left\{{x_{1}}^{3},{x_{2}}^{3},{x_{1}}^{2}x_{2},x_{1}{x_{2}}^{2}\right\}$ in *PseudoKernelSet* based on the kernel function $K\left(x_{1},x_{2}\right)={\left({x_{1}}^{\text{T}}x_{2}\right)}^{3}$. PKF-k-TSP_4_ represents PKF-k-TSP method with the spanned feature space $\left\{1,x_{1},x_{2},{x_{1}}^{2},{x_{2}}^{2},x_{1}x_{2},{x_{1}}^{2}x_{2},x_{1}{x_{2}}^{2},{x_{1}}^{3},{x_{2}}^{3}\right\}$ in *PseudoKernelSet* based on the kernel function $K\left(x_{1},x_{2}\right)={\left(1+{x_{1}}^{\text{T}}x_{2}\right)}^{3}$.

### 3.3 PKF-k-TSP Performance Analysis

To demonstrate the validity of the acceleration algorithm PKF-k-TSP, KF-k-TSP was compared with PKF-k-TSP from muti-views, including the classification performance, time complexity, computation cost, and the selected feature pairs.

#### 3.3.1 Classification performance

Table 4 presents the quantitative results of the ablation study for the proposed PKF-k-TSP framework. To evaluate the contribution of different kernel configurations, we compared the full PKF-k-TSP model against four variants. The experimental results demonstrate that the standard PKF-k-TSP consistently achieves superior or competitive performance across the majority of benchmark datasets. Specifically, PKF-k-TSP reached the highest mean accuracy of 83.79%, outperforming other variants in 5 out of 8 datasets. Moreover, a t-test between PKF-k-TSP and each baseline method is performed, and “*” indicates that the corresponding baseline method significantly outperforms or underperforms PKF-k-TSP at the 0.05 significance level. “Win/Tie/Loss” means that a method has a significantly higher / no significant difference/significantly lower accuracy than PKF-k-TSP across more, an equal number, While certain variants (e.g., PKF-k-TSP_2_ and PKF-k-TSP_4_) showed marginal improvements on specific datasets like *Wdbc* and *Promoter*, the overall p-values suggest that the integration of the proposed components in PKF-k-TSP provides the most robust generalization capability. This confirms that the current architectural design of PKF-k-TSP is optimal for handling high-dimensional biological data.

#### 3.3.2 Time complexity analysis

In addition to the classification performance, time complexity is also a core metric for evaluating algorithms, especially when handling high-dimensional and large-scale datasets. Algorithms with high time complexity often suffer from excessive processing times or even failure to run, severely limiting efficiency. There is one characteristic of omics data, “high dimensions, small samples”, which have a large number of features but few samples. The computation time complexity of KF-k-TSP algorithms increases exponentially with the increase of the feature dimension. To demonstrate the computational efficiency of the acceleration algorithm PKF-k-TSP, the time complexity and the computation costs were shown in Table 5.

Where m denotes the number of samples and n denotes the number of molecular features, the computational complexity reported in Table 5 indicates that PKF-k-TSP incurs substantially lower time costs than both KF-k-TSP and the other PKF-k-TSP variants. On the breast cancer dataset using a five-fold, 50-repeat setting, PKF-k-TSP achieves the shortest runtime (8172 s), reducing computation by 72.43% relative to KF-k-TSP and clearly outperforming all higher-order PKF variants. This efficiency advantage stems from its minimal pseudo-kernel feature space $\left\{{x_{1}}^{2},{x_{2}}^{2},x_{1}x_{2}\right\}$, which effectively avoids the exponential computational burden introduced by higher-order kernel expansions.

Integrating the accuracy and runtime results from Tables 4 and 5, PKF-k-TSP provides the most favorable balance between predictive performance and computational efficiency. Although PKF-k-TSP_4_ achieves slightly higher accuracy, its third-order kernel leads to a substantial increase in computation time, restricting its scalability in high-dimensional omics applications. Accordingly, PKF-k-TSP serves as the recommended default model for large-scale biomedical data analysis or scenarios emphasizing rapid clinical translation, offering strong robustness and excellent scalability.

However, for small-scale validation studies where maximizing accuracy is critical and sufficient computational resources are available, PKF-k-TSP_4_ remains a viable alternative. Intermediate variants such as PKF-k-TSP_2_ and PKF-k-TSP_3_ offer flexible trade-offs between feature richness and computational cost, enabling researchers to adjust kernel complexity according to specific biological hypotheses and resource constraints.

#### 3.3.3 Feature pairs analysis

**Fig. 3:**
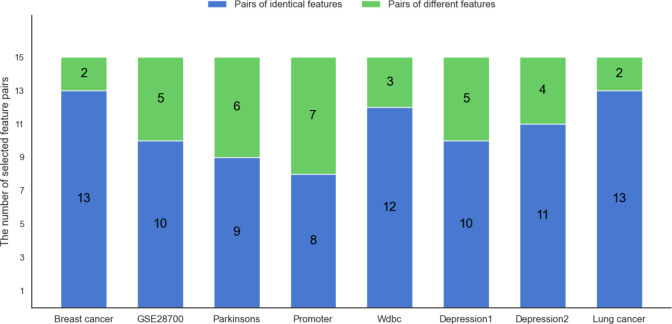
The overlapping results of the feature pairs selected by PKF-k-TSP and KF-k-TSP.

To evaluate the effectiveness of the proposed acceleration algorithm while preserving the core capabilities of the original method, we analyzed the consistency of the feature pairs selected by PKF-k-TSP algorithm and KF-k-TSP algorithm across eight omics datasets. All selected feature pairs are ranked based on their selection frequency, and the top 15 most frequently selected feature pairs are retained for further analysis. Figure 3 shows the overlapping and differential results of the feature pairs selected by PKF-k-TSP and KF-k-TSP. In Figure 3, the blue regions represent the number of the same feature pairs selected by the two algorithms in each omics dataset, while the green regions represent the number of the different feature pairs selected by the two algorithms in each omics dataset. It can be seen from Figure 3 that a large number of feature pairs selected is overlapping in most datasets. For example, in the datasets(*Breast Cancer* and *Lung Cancer*), the number of overlapping feature pairs selected by the two algorithms is 13, accounting for 86.7%. In the datasets(*Parkinsons, Wdbs*, *GSE28700, Depression*1 and *Depression*2), the number of same feature pairs number are 12, 10, 9, 10, and 11 respectively, they all account for over 60%. Only in the dataset *Promoter*, the number of same feature pairs selected by the two algorithms is 8, but the percentage still reach approximately 53.3%, exceeding to 50%. Hence, there is a high consistency in the feature pairs selected by PKF-k-TSP and KF-k-TSP. Based on the above experimental results, the accelerated method PKF-k-TSP significantly reduces computation time while maintaining high overlap with the feature pairs selected by KF-k-TSP algorithm. This high degree of consistency indicates that the acceleration strategy retains the ability to the key feature pairs identified by KF-k-TSP.

### 3.4 Comparison of PKF-k-TSP and compared methods

In this section, the performance of PKF-k-TSP was evaluated on eight public datasets. Meanwhile, PKF-k-TSP was compared with five conversion strategies(sum, diff, sign, mul, abs) and four TSP family algorithms(k-TSP, AUC-TSP, VH-k-TSP, LC-k-TSP) to evaluate effectiveness and adaptability of algorithm.

**Fig. 4:**
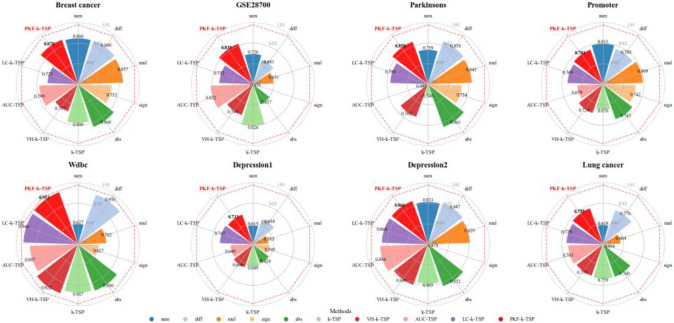
The average classification accuracy rates(%) for each of the eight omics dataset.

Figure 4 presents the comparison of the average classification accuracy rates for each method on each omics dataset. PKF-k-TSP achieves the best classification results on four datasets(*Breast cancer, GSE*28700*, Wdbc*, and *Lung cancer*). Moreover, PKF-k-TSP significantly outperforms sum, diff, sign, mul, abs, k-TSP, AUC-TSP, VH-k-TSP and LC-k-TSP over 9, 9, 7, 6, 9, 8, 8, 9 by at most 24.25%, 84.13%, 73.47%, 15.45%, 51.95%, 45.00%, 80.83%, 60.93%, respectively. Hence, PKF-k-TSP consistently obtains superior or comparable classification accuracy rates across most datasets. Particularly, PKF-k-TSP improves efficiency without sacrificing classification performance or generalizability.

**Fig. 5:**
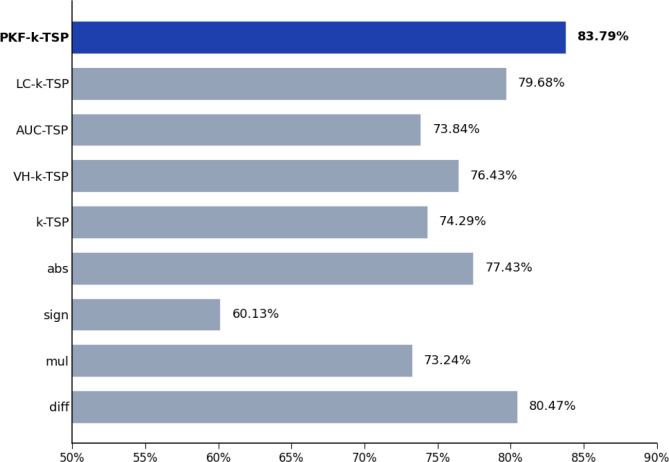
The average classification accuracy rates(%) on the all omics dataset.

Figure 5 presents the comparison of the average classification accuracy rates for each method on all omics dataset. The proposed acceleration algorithm PKF-k-TSP achieves the highest classification accuracy rates(83.79%) and outperforms all baseline methods overall. The average improvement ranges from 3.32% to 23.66%. This further validates that PKF-k-TSP not only improves computational efficiency but also maintains comprehensive and stable classification performance.

Table 6 presents the average classification accuracy of each method across all eight datasets under various odd values of k. The highest accuracy for each k is highlighted in bold. To assess statistical significance, t-tests were conducted between each baseline method and PKF-k-TSP. An asterisk “*” denotes a statistically significant difference (p<0.05), indicating that the baseline method performs significantly worse than PKF-k-TSP. These results demonstrate that PKF-k-TSP not only achieves superior performance but also exhibits strong robustness across a wide range of k values. Additionally, the feature pairs selected by PKF-k-TSP are more informative and discriminative.

In conclusion, PKF-k-TSP efficiently constructs the corresponding feature combinations, accurately measures the interactions between biomolecules, and deeply explores their relationships. It builds models with high accuracy, fast processing speed, and low time complexity, making it particularly advantageous for disease classification and research.

We compared our proposed PKF-k-TSP method with several representative machine-learning and deep-learning approaches, including Multilayer Perceptron (MLP), Elastic Net (EN), SVM, and SVM-RFE. In omics data analysis, SVM is one of the most widely used classification algorithms, whereas SVM-RFE is a popular wrapper-based feature selection method that iteratively removes non-informative features. The Elastic Net integrates the advantages of both L1 and L2 regularization and is particularly suitable for high-dimensional settings with correlated features. In addition, MLP provides a nonlinear deep-learning baseline capable of capturing complex feature interactions in biological datasets. We employed an inner cross-validation loop to determine the optimal value of the parameter k for PKF-k-TSP, searching over the range 1≤k≤15 restricted to odd integers. Since PKF-k-TSP consistently demonstrated superior performance compared with its higher-order PKF family variants, we focus our discussion on this specific model in the subsequent analysis.

The “Avg” in Table 7 is the average accuracy of each method on the eight datasets. Bold font represents the highest value among all the methods on a dataset. “Win/Tie/Loss” represents the number of datasets which has higher (or equal, lower) accuracy compared with PKF-k-TSP for all other methods. Table 7 summarizes the comparison results of PKF-k-TSP against MLP, Elastic Net, SVM, and SVM-RFE. Moreover, a t-test between PKF-k-TSP and each baseline method is performed, and “*” indicates that the corresponding baseline method significantly outperforms or underperforms PKF-k-TSP at the 0.05 significance level. The results show that PKF-k-TSP exhibits outstanding classification accuracy across most datasets. It not only significantly surpasses MLP but is also superior to the Elastic Net (EN), and its performance exceeds that of methods like SVM and SVM-RFE. This performance advantage stems from its adaptability to high-dimensional omics data: MLP may face challenges with insufficient feature representation or overfitting when handling this type of data, while the Elastic Net, as a linear model, struggles to capture complex non-linear feature correlations that are vital for classification accuracy.

However, PKF-k-TSP fundamentally differs from traditional approaches like SVM and SVM-RFE, which primarily rely on single-feature input or selection. The distinction lies in PKF-k-TSP core methodology, which is based on constructing and classifying using feature pairs. Furthermore, PKF-k-TSP belongs to the PKF method family and its performance is superior to its higher-order variants.

In conclusion, PKF-k-TSP efficiently constructs the corresponding feature combinations, accurately measures the interactions between biomolecules, and deeply explores their relationships. By building models with high accuracy, fast processing speed, and low time complexity, PKF-k-TSP proves particularly advantageous for disease classification and research in high-dimensional omics settings.

## 3.5 Biomolecules selected by PKF-k-TSP

This section focuses on analyzing the metabolite pairs selected by the PKF-k-TSP on the lung cancer dataset and exploring the corresponding biomedical meaning. Table 8 lists the top nine representative metabolite pairs and their corresponding combination functions based on PKF-k-TSP. The top-nine pairs contain 10 different metabolites.

**Fig. 7:**
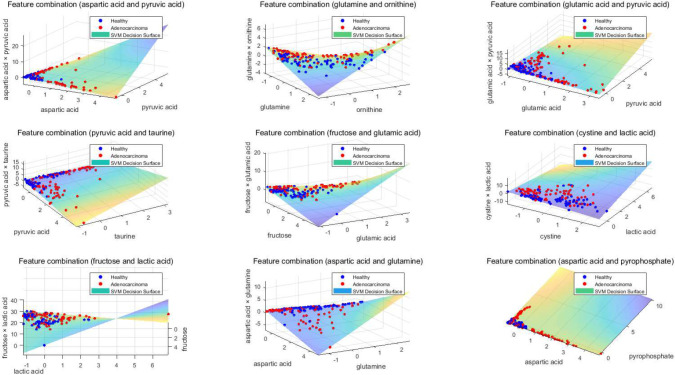
Combination forms of the nine metabolite pairs and cut-off rules.

We performed the pathway analysis to explore the functions of the metabolites. Figure 7 shows the results of metabolic pathway enrichment analysis, highlighting several significantly enriched pathways, such as Arginine biosynthesis, Alanine, aspartate and glutamate metabolism, Glyoxylate and dicarboxylate metabolism, Citrate cycle (TCA cycle), Phenylalanine metabolism, etc. Among them, the pathway with the most significant enrichment is related to lung cancer. arginine biosynthesis exhibited the highest statistical significance (-log10(p) > 8), suggesting its potential role as a core regulatory pathway in disease progression. Previous studies have also shown that MYC-driven small cell lung cancer depends on arginine metabolism, and arginine deprivation therapy can effectively inhibit tumor growth (Chalishazar et al. 2019).

In addition to biological relevance, we further examined the selection frequency of metabolite pairs across repeated cross-validation runs. Several key metabolite pairs, including aspartic acid–pyruvic acid and glutamine–ornithine, were consistently selected in more than 80% of runs, indicating strong robustness against data resampling. This high recurrence suggests that these metabolite interactions represent stable disease-associated patterns rather than dataset-specific artifacts.

Figure 7 shows the combination patterns of nine key metabolite pairs and their corresponding cut-off rules determined by the PKF-k-TSP method. In the three-dimensional (3D) feature space, a Support Vector Machine (SVM) decision surface is used to distinguish between the healthy and lesion group (Demidova 2024). Seven metabolite pairs (aspartic acid–pyruvic acid, glutamic acid–pyruvic acid, pyruvic acid–taurine, fructose–glutamic acid, cystine–lactic acid, fructose–lactic acid, and aspartic acid–glutamine) exhibit approximately linear separation. The data points of the two groups are distributed on either side of the SVM surface, making them promising discriminative features. In contrast, two metabolite pairs (aspartic acid–pyrophosphate and glutamine–ornithine) display more complex and potentially non-linear distributions relative to the SVM surface. For instance, the glutamine–ornithine pair, which is involved in the arginine biosynthesis pathway, may exhibit such complexity due to mutual conversion via biochemical reactions. Overall, Figure 7 visually demonstrates the separation ability of the SVM decision surface and highlights the biological interpretability and biomarker potential of the metabolite pairs selected by the PKF-k-TSP method.

A key characteristic of tumor metabolism is metabolic reprogramming. In line with the Warburg effect (Liberti and Locasale 2016), cancer cells preferentially enhance aerobic glycolysis of glucose, relying on the glycolytic pathway for energy production even under oxygen-rich conditions. This enables them to meet the metabolic demands required for rapid proliferation and survival. Notably, the PKF-k-TSP method employs a polynuclear function strategy, allowing for a comprehensive exploration of the complex combinatorial relationships between metabolites from both a higher-dimensional and nonlinear perspective. This approach offers greater flexibility and accuracy compared to traditional linear methods. It not only improves the ability to identify metabolic differences across various disease states but also provides robust support for a deeper understanding of the metabolic regulatory mechanisms in tumors.

### 3.6 Pan-cancer Analysis via PKF-k-TSP

To investigate the commonalities and specificities of oncogenic mechanisms across multiple cancer types, we selected six representative cancers from The Cancer Genome Atlas TCGA(http://xena.ucsc.edu) database—BLCA, BRCA, CHOL, KICH, KIRC, and READ—for a comprehensive multi-omics integration analysis. To precisely identify key drivers from the high-dimensional data, we employed the PKF-k-TSP algorithm to select the top 15 pathway interaction pairs with high discriminative power for each cancer type. This strategy effectively filters stochastic noise across multi-omics platforms while ensuring the biological “functional relevance” of the selected pathways, thus establishing a solid foundation for subsequent analysis of pan-cancer common mechanisms and identification of tissue-specific signaling networks.

**Fig. 8:**
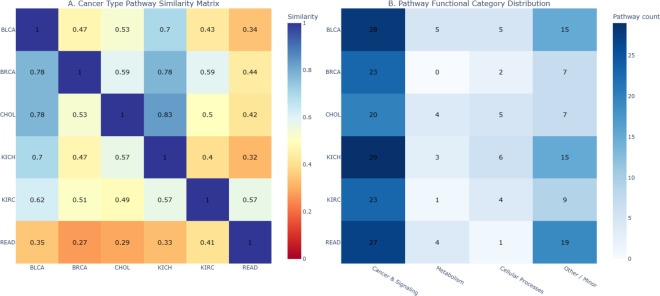
The Landscape of Pathway Similarities and Enrichment in Cancers.

**Fig. 9:**
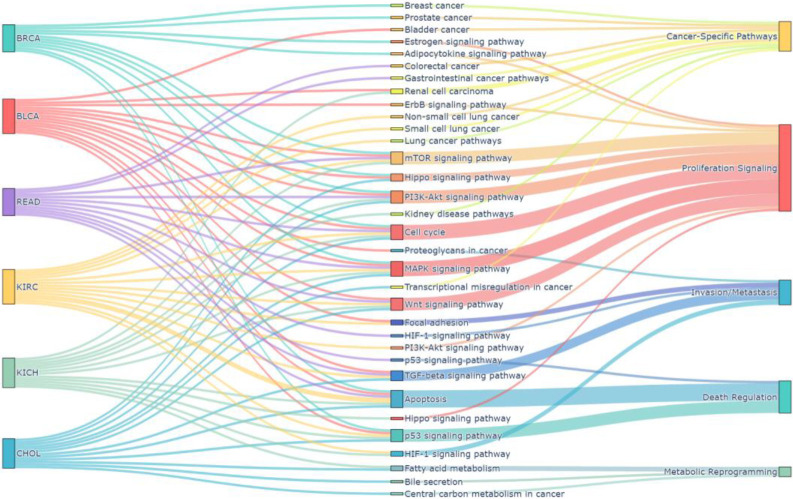
Characteristic Signaling Pathways.

A quantitative comparison of pathway profiles across cancer types using Jaccard similarity coefficients clearly revealed molecular kinship among cancers (Figure 8A) (Bursalioglu et al. 2025). BLCA and KICH exhibited the highest similarity, with substantial overlap in pathways regulating cell polarity and microenvironment remodeling, such as Hippo and TGF-β, reflecting shared tissue origins within the urinary system at the molecular level. In contrast, READ displayed a distinct pathway landscape, highlighting the unique oncogenic trajectory dominated by dysregulation of the Wnt/APC axis in colorectal cancer (Hoadley et al. 2018). This similarity pattern demonstrates the algorithm's capability to capture essential disease connections. Quantitative analysis of Figure 8B revealed that all six cancer types exhibited their most prominent enrichment in the “Cancer & Signaling” category, with pathway counts ranging from 20 to 29 per cancer type, underscoring the pivotal role of signaling dysregulation in pan-cancer progression (Hanahan and Weinberg 2011). Notably, CHOL and KICH demonstrated unique metabolic reprogramming signatures (containing 4 and 3 metabolism-related pathways, respectively), while BRCA showed no such enrichment. This tissue specificity is further dissected in the Sankey diagram (Figure 9), which distinguishes between the two renal subtypes: KICH is characterized by fatty acid metabolism consistent with mitochondrial dysfunction, whereas KIRC exhibits a stronger enrichment in angiogenesis signaling (e.g., the HIF-1 pathway) reflecting its hallmark VHL gene loss.

To further dissect the hierarchical structure and functional mapping, a three-dimensional Sankey diagram of “Cancer–Pathway–Function” was constructed (Figure 9). This visualization elucidates both universal pan-cancer patterns and fine-grained tissue-specific signatures (Sanchez-Vega et al. 2018). At the functional level, core hub pathways—such as PI3K-Akt and MAPK—traverse all six cancers like “main arteries,” while the prevalence of the p53 apoptosis pathway validates the ubiquity of cell cycle dysregulation across oncogenesis. At the tissue-specific dimension, the diagram illustrates distinct connection patterns. Within the urinary system, BLCA is extensively linked to both proliferation and metastasis networks, reflecting its dual malignant characteristics. Notably, our study incorporates two subtypes of common renal origin: KICH and KIRC. The Sankey diagram reveals that KICH (Davis et al. 2014) is characterized by unique metabolic reprogramming signatures, particularly in fatty acid metabolism, consistent with clinically observed mitochondrial dysfunction. In contrast, KIRC exhibits a stronger enrichment in angiogenesis and pro-invasive signaling (e.g., the HIF-1 pathway) (The Cancer Genome Atlas Research Network 2013), accurately reflecting the molecular hallmark of VHL gene loss. Furthermore, the strong association between BRCA (The Cancer Genome Atlas Network 2012) and the estrogen signaling pathway corresponds to its hormone-dependent molecular subtype. Intriguingly, the convergence of invasion-related pathways in BLCA, LIRC, and READ aligns with their high clinical metastatic propensity. This hierarchical mapping from “central hubs” to “tissue branches” underscores the robustness of the PKF-k-TSP algorithm in capturing subtle molecular distinctions even between subtypes of the same organ, thereby confirming its profound clinical relevance.

In summary, the pathways identified in this study not only exhibit significant statistical robustness but also provide profound biological insights, they offer a molecular basis for “treating different diseases with the same therapy” across cancer types (Reel et al. 2021) and present a clear roadmap for precision medicine. By integrating multi-omics data through the PKF-k-TSP algorithm, this study successfully constructs a multi-layered signaling network framework, providing a powerful analytical tool for future research in capturing both pan-cancer patterns and subtle tissue specificity.

## 4. Discussion

This study evaluated the effectiveness of the accelerated algorithm PKF-k-TSP for multi-view bioinformatics datasets. Comprehensive comparisons show PKF-k-TSP has significant advantages in classification performance, time complexity, and feature selection. PKF-k-TSP was developed to address limitations of traditional kernel-based methods: polynomial and RBF kernels have prohibitive computational burden for large-scale omics data and risk overfitting, reducing interpretability. PKF-k-TSP uses explicit pseudo-kernel mapping to approximate kernel transformations, drastically reducing complexity while capturing linear and nonlinear feature relationships, making it suitable for high-dimensional omics data.

Our findings show PKF-k-TSP classification accuracy is superior or on par with other algorithms. On the *Breast cancer* dataset, it had excellent speed and cross-validation confirmed reduced runtime without sacrificing performance. PKF-k-TSP also excels in feature selection by identifying discriminative combinations, such as nine key lung cancer metabolite pairs including aspartic acid/pyruvic acid, which helps elucidate tumor metabolic reprogramming related to the Warburg effect. It also performs well in pan-cancer analysis, capturing shared signaling hubs and tissue-specific networks across six TCGA cancer types, with polynomial pseudo-kernels enabling the modeling of complex relationships.

PKF-k-TSP complements rather than replaces large-scale integration frameworks, targeting high-dimensional, small-sample scenarios that require transparent rules. The current study has limitations, including the use of a limited number of datasets that require broader validation, and metabolite findings that need further experimental and clinical translation (Karczewski and Snyder 2018).

In conclusion, PKF-k-TSP is an efficient, accurate, and interpretable algorithm that combines the interpretability of TSP-family methods with the efficiency of pseudo-kernel mappings. Future work may integrate PKF-k-TSP with other machine learning frameworks to address more complex biological questions and accelerate the discovery and clinical application of biomarkers.

## 5. Conclusion

This paper proposes a novel omics data analysis method based on pseudo kernel functions to speed up the discovery of feature combinations, PKF-k-TSP. PKF-k-TSP maps feature pairs from a low-dimensional space to a high-dimensional feature space by a mapping function, constructs feature combinations in new high-dimensional feature space, and selects k>0 top-scoring pairs to build an ensemble classifier. The results from four aspects(classification accuracy rate, time complexity, computation cost and the selected feature pairs) between PKF-k-TSP and KF-k-TSP shows that the accelerated algorithm PKF-k-TSP maintains the quality of selected feature pairs and classification performance while significantly reducing time complexity and computational cost. The results between PKF-k-TSP and other comparison methods shows that PKF-k-TSP is superior to sum, diff, sign, mul, abs, k-TSP, AUC-TSP, VH-k-TSP and LC-k-TSP in most cases. Meanwhile, the PKF-k-TSP method demonstrates superior classification performance over traditional and baseline models, including SVM, SVM-RFE, EN, and MLP. Furthermore, the feature pairs defined by PKF-k-TSP possess explicit biological significance. Crucially, our pan-cancer analysis demonstrates the algorithm's ability to distinguish conserved oncogenic hubs from tissue-specific metabolic and pro-invasive signatures. Hence, PKF-k-TSP effectively improves computational efficiency while maintaining classification performance, which provided an effective tool for large-scale disease omics data analysis and uncovering latent biological mechanisms.

## Figures and Tables

**Fig. 6:**
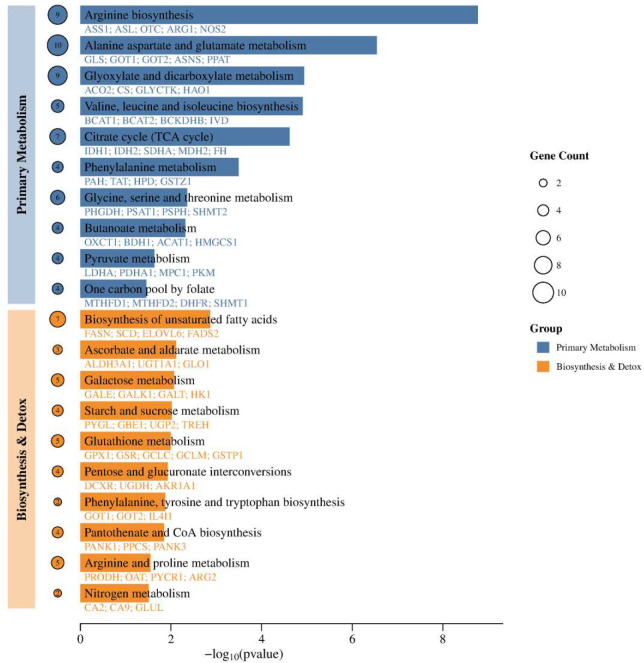
Pathway enrichment analysis.

**Table 1.** Statement of Significance

Problem or Issue	Discovering interpretable molecule combinations for cancer classification is vital for diagnosis and prognosis, but existing methods have prohibitive computational complexity for high-dimensional omics data, hindering cancer analysis.
What is Already Known	Molecule combinations aid disease and cancer analysis; methods like KF-k-TSP explore combinations but have high computational costs for large-scale omics data, limiting application.
What This Paper Adds	This study introduces a novel acceleration algorithm and a pseudo kernel function-based omics data analysis framework (PKF-k-TSP) for disease and cancer analysis. By mapping feature pairs to a high-dimensional space via pseudo kernel functions, the approach demonstrates superior classification performance while reducing computational time by 72.43% compared with KF-k-TSP, preserving interpretability through biologically meaningful feature pairs and supporting cross-cancer pathway interaction analysis.

**Table 2.** Eight public datasets.

Dataset	Features	Samples	Classes	Source
Breast cancer	162	271	2	(Budczies et al. 2013)
GSE28700	556	44	2	(Tseng et al. 2011)
Parkinsons	22	195	2	(Elghazel and Aussem 2015; Markelle et al. 2023)
Promoter	57	106	2	(Elghazel and Aussem 2015; Markelle et al. 2023)
Wdbc	30	569	2	(Elghazel and Aussem 2015; Markelle et al. 2023)
Depression1	143	97	2	--
Depression2	391	94	2	--
Lung cancer	152	172	2	--

**Table 3.** The detailed descriptions of kernel functions and mapping functions

Kernel Functions	Mapping functions	Name
$k_{1}\left(x_{1},x_{2}\right)={\left(x_{1}^{\text{T}}x_{2}\right)}^{2}$	$\phi_{1}\left(x_{1},x_{2}\right)=\left\{x_{1}^{2},x_{2}^{2},x_{1}x_{2}\right\}$	PKF-k-TSP_1_
$k_{2}\left(x_{1},x_{2}\right)={\left(1+x_{1}^{\text{T}}x_{2}\right)}^{2}$	$\phi_{2}\left(x_{1},x_{2}\right)=\left\{1,x_{1},x_{2},x_{1}x_{2},x_{1}^{2},x_{2}^{2}\right\}$	PKF-k-TSP_2_
$k_{3}\left(x_{1},x_{2}\right)={\left(x_{1}^{\text{T}}x_{2}\right)}^{3}$	$\phi_{3}\left(x_{1},x_{2}\right)=\left\{x_{1}^{3},x_{2}^{3},x_{1}^{2}x_{2},x_{1}x_{2}^{2}\right\}$	PKF-k-TSP_3_
$k_{4}\left(x_{1},x_{2}\right)={\left(1+x_{1}^{\text{T}}x_{2}\right)}^{3}$	$\phi_{4}\left(x_{1},x_{2}\right)=\left\{1,x_{1},x_{2},x_{1}x_{2},x_{1}^{2},x_{2}^{2},x_{1}^{2}x_{2},x_{1}x_{2}^{2},x_{1}^{3},x_{2}^{3}\right\}$	PKF-k-TSP_4_

**Table 4.** Ablation study of PKF-k-TSP variants in terms of classification accuracy (%).

Dataset	PKF-k-TSP	PKF-k-TSP_1_	PKF-k-TSP_2_	PKF-k-TSP_3_	PKF-k-TSP_4_
Breastcancer	**87.82**	76.09*	87.30*	85.63*	86.37*
GSE28700	**83.89**	41.98*	76.23*	72.04*	77.57*
Parkinsons	85.80	82.42*	85.96*	87.04*	87.46*
Promoter	78.09	78.25*	79.64	77.55*	**80.81**
Wdbc	95.29	77.87*	**95.30**	93.06*	95.26
Depression1	**73.27**	54.64*	69.70*	65.31*	70.51*
Depression2	**86.60**	81.18*	85.08	84.39	83.60
Lung cancer	**79.55**	76.65*	78.09*	75.76*	76.26*
avg	83.79	71.14	82.16	80.10	82.23
Win/Tie/Loss		1/0/7	1/3/4	1/1/6	1/3/4

**Table 5.** Comparison of time Complexity.

Method	Time Complexity	Computation Costs
PKF-k-TSP	$\text{O}\left(m^{2}n^{2}\right)$	8172s
PKF-k-TSP_1_	$\text{O}\left(2*m^{2}n^{2}\right)$	10052s
PKF-k-TSP_2_	$\text{O}\left(2*m^{2}n^{2}\right)$	12129s
PKF-k-TSP_3_	$\text{O}\left(4*m^{2}n^{2}\right)$	55138s
PKF-k-TSP	$\text{O}\left(6*m^{2}n^{2}\right)$	56731s
KF-k-TSP	$\text{O}\left(4*m^{2}n^{2}\right)$	29644s

**Table 6.** The average classification accuracy rates(%) for each k on all the datasets.

Method	k=1	k=3	k=5	k=7	k=9	k=11	k=13	k=15
sum	72.44*	73.36*	73.36*	73.29*	73.35*	73.30*	72.90*	72.66*
diff	78.04*	79.81*	80.21*	80.52*	80.98*	81.43*	80.76*	80.73*
mul	70.58*	72.15*	72.29*	72.57*	73.48*	74.31*	73.58*	74.33*
sign	58.72*	59.76*	60.07*	60.26*	60.45*	60.56*	58.49*	58.49*
abs	75.88*	76.51*	77.19*	77.43*	77.90*	78.12*	77.11*	77.09*
k-TSP	73.68*	74.39*	74.12*	74.47*	73.60*	74.43*	77.87*	77.76*
VH-k-TSP	76.52*	77.14*	77.12*	76.17*	76.18*	75.93*	76.36*	76.03*
AUC-TSP	73.58*	74.61*	74.17*	74.75*	73.18*	71.41*	78.26*	77.94*
LC-k-TSP	82.07*	82.40*	82.38*	81.89*	81.14*	75.66*	77.02*	74.02*
PKF-k-TSP	**86.94**	**88.34**	**87.88**	**87.88**	**87.94**	**88.01**	**87.99**	**87.92**

**Table 7.** Comparison in accuracy (%) among PKF-k-TSP, SVM, SVM–RFE, EN and MLP.

Dataset	SVM	SVM-RFE	EN	MLP	PKF-k-TSP
Breastcancer	87.36	82.14*	84.52*	80.64*	**87.82**
GSE28700	73.76*	69.64*	89.14*	48.41*	83.89
Parkinsons	**86.22**	85.10	79.85*	82.64*	85.80
Promoter	76.43*	76.00*	72.73*	56.67*	**78.09**
Wdbc	97.28*	96.63*	97.35*	99.12*	95.29
Depression1	79.20*	77.55*	76.41*	56.45*	73.27
Depression2	86.56	**87.43**	86.93*	66.78*	86.60
Lung cancer	78.65*	78.12*	78.54*	74.42*	**79.55**
avg	82.73	81.58	83.18	70.64	**83.79**
Win/Tie/Loss	2/3/3	2/2/4	4/0/4	1/0/7	

**Table 8.** The top-nine metabolite pairs selected by PKF-k-TSP

Pair no.	Metabolite 1	Metabolite 2	Combination functions
1	Aspartic acid	Pyruvic acid	−0.78 × Aspartic acid + 0.45 × Pyruvic acid + 0.35 × Aspartic acid*Pyruvic acid +0.00
2	Glutamine	Ornithine	−0.70 × Glutamine − 1.11 × Ornithine + 0.72 × Glutamine*Ornithine −0.39
3	Glutamic acid	Pyruvic acid	−0.66 × Glutamic acid + 0.55 × Pyruvic acid + 0.73 × Glutamic acid*Pyruvic acid + 0.10
4	Pyruvic acid	Taurine	0.57 × Pyruvic acid − 0.90 × Taurine + 0.99 × Pyruvic acid*Taurine + 0.22
5	Fructose	Glutamic acid	−0.90 × Fructose − 0.87 × Glutamic acid − 0.30 × Fructose*Glutamic acid − 0.06
6	Cystine	Lactic acid	−1.65 × Cystine − 0.63 × Lactic acid + 0.27 × Cystine*Lactic acid − 0.22
7	Fructose	Lactic acid	−0.97 × Fructose − 1.08 × Lactic acid − 0.38 × Fructose*Lactic acid − 0.12
8	Aspartic acid	Glutamine	−0.81 × Aspartic acid − 0.89 × Glutamine +0.46 × Aspartic acid*Glutamine − 0.09
9	Aspartic acid	Pyrophosphate	−1.07 × Aspartic acid +0.54 × Pyrophosphate −0.04 × Aspartic acid*Pyrophosphate − 0.11

## Data Availability

The datasets analysed in this study are publicly available. The GSE28700 dataset is available from the Gene Expression Omnibus (GEO) database. The Parkinsons, Promoter, and Wdbc datasets are available from the UCI Machine Learning Repository. The Depression1, Depression2, and Lung cancer datasets are available from the Metabolomics Workbench under project IDs ST000062, ST000063, and ST000385, respectively. The TCGA pan-cancer datasets used for pathway interaction analysis are available from the UCSC Xena platform. The Breast cancer dataset was obtained from the supplementary materials of the previously published study cited in the manuscript.

## Code Availability

The source code used in this study is available from the corresponding author upon reasonable request.
